# Moral Violations and the Experience of Disgust and Anger

**DOI:** 10.3389/fnbeh.2018.00179

**Published:** 2018-08-22

**Authors:** Megan Oaten, Richard J. Stevenson, Mark A. Williams, Anina N. Rich, Marina Butko, Trevor I. Case

**Affiliations:** ^1^Menzies Health Institute Queensland, Griffith University, Southport, QLD, Australia; ^2^Department of Psychology, Macquarie University, Sydney, NSW, Australia; ^3^Department of Cognitive Science & ARC Centre for Cognition and its Disorders, Macquarie University, Sydney, NSW, Australia; ^4^Perception in Action Research Centre, Macquarie University, Sydney, NSW, Australia

**Keywords:** disgust, moral disgust, moral violations, anger, fMRI

## Abstract

Disgust is a natural defensive emotion that has evolved to protect against potential sources of contamination and has been recently linked to moral judgements in many studies. However, that people often report feelings of disgust when thinking about feces or moral transgressions alike does not necessarily mean that the same mechanisms mediate these reactions. The present study used functional magnetic resonance imaging (*n* = 22) to investigate whether core and moral disgusts entrain common neural systems. We provide evidence that: (i) activation of overlapping brain regions between core and moral disgust is the result of content overlap in the vignettes—*core disgust elicitors*—across conditions, and not from moral violations *per se*, and (ii) moral residue (i.e., the remaining or “residual” activation after the influence of core disgust elicitors have been taken into account) produced a pattern of activation that is more consistent with moral anger, than one of “residual disgust.” These findings run contrary to the premise that our “moral center” is connected to the area of the brain in which physical revulsion is located.

## Introduction

The emotion of disgust is typically experienced as a feeling of revulsion elicited by something offensive—e.g., bodily fluids and waste, animal products, rotten food, and certain classes of sexual behavior, and is accompanied by a strong desire to withdraw from the eliciting stimulus (Rozin et al., 1984; Oaten et al., 2009). More recently, researchers have found that participants tested across a number of experimental paradigms report feelings of disgust when considering both offensive substances (e.g., core disgust—feces, vomit, blood, and rotten meat) and immoral behavior (e.g., moral disgust—incest, murder; Haidt et al., 1997; Lieberman et al., 2003), suggestive of a link between disgust and morality (Schaich Borg et al., 2008). It has been further suggested that disgust may be linked to “purity transgressions”—essentially moral violations that contain a core disgust referent—and not necessarily other types of moral violations (e.g., moral violations that do not contain a core disgust referent; Horberg et al., 2009). This is an interesting point that we return to shortly. The neural signatures produced by core disgust stimuli vs. moral disgust stimuli (i.e., involving moral infractions) are purported to entrain substantial overlap (Moll et al., 2005; Schaich Borg et al., 2008). Despite emerging consensus for the view that our “moral center” is connected to the area of the brain in which physical revulsion is mediated (Haidt, 2001; Schaich Borg et al., 2008; Schnall et al., 2008), we argue that the support for this premise is problematic. In particular, it remains unclear whether the overlap of neural activation between core and moral disgust is limited to those moral transgressions, as noted above, simply by virtue of their reference to core disgust elicitors (e.g., feces, vomit, blood, and rotten meat), or whether the presence of a moral transgression generates *additional* disgust, and hence greater activation, to that produced by a matched core disgust condition free of moral connotation—thereby establishing a “true” moral disgust.

Disgust is considered to be distinguishable from other emotive responses—e.g., fear (Phillips et al., 1997; Wicker et al., 2003), and anger (Sprengelmeyer et al., 1998)—and has been associated with activity in the anterior insula, basal ganglia, medial prefrontal cortex, thalamus, and visual cortex (Sprengelmeyer et al., 1998; Wicker et al., 2003; Moll et al., 2005; Williams et al., 2005; Schaich Borg et al., 2008; Schnall et al., 2008), in both clinical populations with impaired disgust processing [e.g., neurological (Hayes et al., 2007) and psychiatric (Vicaro et al., 2017) conditions] and non-clinical populations (Moll et al., 2005; Schaich Borg et al., 2008). However, investigation of the neural correlates of disgust in the context of moral behavior is limited. A widely cited fMRI study suggests that reading about certain socio-moral violations can induce patterns of neural activity that overlap with those observed when reading about more concrete disgust elicitors (Moll et al., 2005). This article is influential because of its claim that socio-moral violations *cause* a disgust response. However, the claim is significantly weakened by two shortcomings in the study design. First, the socio-moral elicitors contain references to core disgust elicitors (e.g., death, spiders, cockroaches, etc). This undermines the claim that it is the socio-moral violation *per se* that *causes* disgust. An equally plausible alternative is that disgust is caused by the reference to the core disgust referents contained within the stimuli (Case et al., 2012). This limitation is not restricted to the work of Moll et al. (2005). Schaich Borg et al.'s (2008) fMRI study presented men with vignettes describing various acts involving their sisters. Although the authors reported overlap in the brain regions activated for core (or in that work, “*pathogen*”) and socio-moral disgusts, the items used to generate these conditions incorporated vivid core disgust referents—i.e., “sipping your sister's urine” (pathogen disgust), “watching your sister masturbate” (sexual moral disgust), and “killing your sister's child (non-sexual moral disgust). That is, in addition to the moral violation, *all* of the vignettes would also bring to mind core disgust elicitors. For example, “killing your sister's child” would very likely bring to mind images of blood and gore, which are quintessential core disgust elicitors. Thus again, brain activation overlap might be due to the overlap in the content of stimulus items—*core elicitors*—across conditions, and not to moral disgust *per se*.

A second shortcoming in experimental design was that neither study contained a matched disgust control condition (e.g., that mentions death, spiders, cockroaches, urine, masturbation, etc); so we do not know what neural structures are routinely activated under conditions where one is simply asked to read vignettes containing disgust-related words drawn from the socio-moral stimuli but without any moral connotations. Relatedly, the absence of a moral anger control condition is noteworthy. Nabi (2002) has argued that the self-reported “disgust” which arises in response to moral offenses is only a metaphorical use of disgust to describe angering situations. Indeed, anger is a plausible response to norm violations or to deception and abuse (Gutierrez and Giner-Sorolla, 2007; Olatunji et al., 2016), and is considered to be responsive to contextual cues of harm and intentionality (Gutierrez and Giner-Sorolla, 2007). Moll et al.'s (2005) socio-moral statements were associated with *both* self-reported disgust and anger, yet the authors did not investigate whether socio-moral violations and anger activate common brain networks, as they did for disgust. It is therefore possible that unmeasured affective activations, separate from the induced disgust, are doing some of the work in driving participants' reactions to moral transgressions (Russell and Giner-Sorolla, 2011). The co-occurrence of anger and disgust in moral situations (Gutierrez and Giner-Sorolla, 2007; Olatunji et al., 2016) suggests that any investigation of the neural correlates of disgust and morality should be extended to also include anger.

We suggest that a strong demonstration of moral disgust—one removed of any core disgust reference—would then need six conditions: moral disgust vignettes, moral anger vignettes (i.e., to identify activation under conditions of anger-provoking stimuli), matched disgust vignettes (i.e., to control for the influence of the core disgust referent contained in the moral disgust vignettes), high disgust vignettes (i.e., to ensure a condition that exceeds the matched disgust vignettes in disgust intensity and to check for activations akin to those observed before for this emotion; Phillips et al., 1997; Sprengelmeyer et al., 1998; Wicker et al., 2003; Moll et al., 2005; Williams et al., 2005; Schaich Borg et al., 2008), neutral control vignettes (i.e., word reading controls) and scrambled vignettes (i.e., task attention controls). To convincingly establish that moral violations generate (moral) disgust, the following would need to be demonstrated: (i) Moral disgust vignettes produce activation at locations that overlap with those activations produced by the high disgust vignettes, *after* contrasting out the influence of the matched core referent word (i.e., moral disgust—matched disgust); and (ii) Moral disgust vignettes exceed activation at key sites (i.e., those implicated *a priori* as being indicative of disgust emotional processing) relative to the matched disgust vignettes. This pattern would show that moral disgust vignettes could induce disgust-like patterns of neural activity independent of core disgust references within the vignette. In addition, it would also show that moral disgust vignettes could induce greater activation relative to the effects of their core referent word.

## Pilot study

The purpose of the pilot work was to generate and evaluate a series of vignettes for the main study to ensure that they differed as expected on a range of characteristics including ratings of emotion, morality, intentionality, and wrongness. We also included ratings of purity, harm, and justice due to the suggestion that moral disgust is reserved for transgressions that are related to “purity violations” (e.g., misuse of the body, etc.), while anger is related to violations of individual rights and community norms (Haidt and Graham, 2007; Landy and Goodwin, 2015). The vignette sets were moral disgust vignettes, moral anger vignettes, matched disgust vignettes that contained the same core disgust referents as the moral disgust vignettes, high disgust vignettes that were meant to induce greater disgust than the matched disgust vignettes, neutral control vignettes and scrambled vignettes (see Table 1).

**Table 1:** The vignettes evaluated in piloting and used in the main study.

*Moral disgust vignettes*
**1.** A man with body odor enjoys grossing out strangers with his smell. **2.** Someone deliberately drops a cockroach on a person's plate of food. **3.** Someone intentionally soils a toilet seat in a public restroom. **4.** A woman enjoys eating chocolate shaped like dog-poo. **5.** A man likes to urinate in public places. **6.** Someone leaves a dead rat in their neighbor's kitchen. **7.** A teacher gets a sexual thrill from inflating a condom, as part of a physical health class. 8. A woman deliberately goes to work in a restaurant when she has influenza. **9.** A person enjoys watching child pornography on the Internet. **10.** A woman finds her apple is rotten and so gives it to her child. **11.** A person intentionally dumps their garbage in the street. **12.** A hotel owner will not fumigate bug-infested beds because it is too expensive. **13.** A young man likes to listen to his elderly neighbors having sex. **14.** A man can't be bothered to wash his hands before preparing his baby's bottle. **15.** A woman forces her kids to clean her mold-infested fridge because she can't be bothered. **16.** A woman is too lazy to clean her teeth and so stinks of bad breath. **17.** A person purposely drops a snot covered tissue in a doctors waiting room. 18. Someone deliberately farts in a grocery queue. **19.** A man intentionally urinates on a toilet floor. **20.** A man lets his dog poo in the kid's sandpit.
*Matched disgust vignettes*
**1.** Someone smells of body odor on a packed bus during rush hour. **2.** A cockroach runs across someone's plate of food. **3.** Someone sits on a dirty toilet seat in a public restroom. **4.** A shop sells chocolates shaped like dog-poos. **5.** A man walks through an underpass smelling of urine. **6.** A dead rat is found lying on someone's kitchen floor. 7. A teacher inflates a new condom, using her mouth, as part of a physical health class. **8.** A woman unknowingly goes to work in a restaurant when she has influenza. **9.** A person accidentally accesses child pornography on the Internet. 10. A woman bites into a rotten apple and quickly disposes of it. 11. A neighborhood's garbage is strewn all over the street. **12.** A person finds their hotel room bed and furniture infested with bed bugs. 13. An elderly neighbor has sex very loudly every night in the apartment next door. 14. A man does not wash his dirty hands before cooking his own evening meal. **15.** A woman opens her fridge and sees mold growing on the sides and shelves. 16. Someone with bad breath smells out the elevator. **17.** A person carries a tissue containing snot in their pocket. **18.** Someone farts in a busy supermarket queue. **19.** A man accidentally urinates on the toilet floor. **20.** Someone sees dog poo in a kid's sandpit.
*High disgust vignettes*
**1.** A woman vomits profusely. **2.** A person changes their underwear only once a week. **3.** Someone's intestines are exposed after being involved in a car accident. **4.** A woman smells urine and vomit when walking through a tunnel under a railroad track. **5.** A man has an attack of diarrhea in a public toilet. **6.** Someone sees maggots on a piece of meat in an outdoor garbage pail. **7.** A person is about to drink a glass of milk when they smell that it is spoiled. **8.** Someone accidently touches a dead body. **9.** A man walks barefoot on the grass and steps in fresh dog feces. **10.** A person's hand is covered in blood after accidentally cutting their finger. **11.** A woman drank from a glass of water containing another person's saliva. **12.** A person accidentally sneezes all over another passenger on a train. **13.** A woman does not wash her hands after using the toilet. **14.** A person runs for the bathroom with diarrhea and didn't make it. **15.** Someone finds their hotel bed stinks of stale urine. **16.** A man finds a cockroach in his meat pie. **17.** A person's bone is sticking out of their leg after an industrial accident. 18. Someone finds a discarded syringe and needle in a kid's sandpit. **19.** A man sits on a public toilet and finds the seat has feces on it. **20.** Someone leaves a soiled tampon on the toilet floor.
*Moral anger vignettes*
**1.** A woman is too busy to help a lost child in a supermarket. **2.** A woman is rude to a Christmas charity collector. **3.** A student refuses to lend her notes to a friend who has missed a lecture. **4.** A manager makes fun of his secretary's new outfit in front of other staff. **5.** Someone pushes ahead of people in a long queue because they are in a hurry. **6.** A shop assistant talks on the phone while people queue to be served. **7.** A woman steals the change from a busker's cap. **8.** A waiter deliberately avoids serving a group of Indian customers. **9.** Some guests in a restaurant shout and jeer ruining everyone else's meal. **10.** A woman steals money from a church collection box. **11.** A student who offered to submit his friend's assignment, copies it before handing it in. **12.** Someone in a block of units plays loud music while everyone else tries to sleep. **13.** A shop assistant serves his friend first while others have to wait. **14.** A wealthy man hires a top lawyer to get off a dangerous driving charge. **15.** A foreign company saves money by leaving out an essential safety feature—but only on exported goods. **16.** A manager puts down his female colleague but only when she is not around. **17.** A telemarketing company will not allow its workers any breaks. 18. A clothing company knowingly sells goods made with child labor. **19.** Someone drives on the wrong side of the road to jump the queue in a traffic jam. **20.** A thief targets the handbags of women busy with young children.
*Neutral vignettes*
**1.** A man catches the train to work. **2.** A woman goes to a restaurant for dinner. **3.** A person goes to the store to buy groceries. **4.** A person catches the bus to the city on the weekend. **5.** A person makes their bed of a morning. **6.** A person eats a bar of chocolate. **7.** A person catches the elevator to the top floor of a building. **8.** A person cooks dinner at home. **9.** A person eats an apple for breakfast. **10.** A woman goes to the movies.
*Scrambled vignettes*
**1.** Catches to man train work the a. **2.** Goes for restaurant woman dinner a a to. **3.** To groceries goes buy store a to the person. **4.** The bus weekend on catches to person the a city. **5.** Their a bed morning of a makes person. **6.** Of man eats chocolate a a bar. **7.** Catches elevator a of top building to a man the floor. **8.** Dinner a home person cooks at.**9.** A man an breakfast eats apple for. **10.** A movies woman the goes to.

Obtaining such self-report data from participants in the main study would be problematic because we wanted scanning participants simply to read the vignettes and react to them without biasing them toward particular aspects of each vignette, such as whether they were disgusting or immoral. We also did not get ratings from these participants before or after the main study because ratings under these conditions would either influence or be influenced by presentation during scanning. If administered before the task, it would have habituated the vignettes' elicitation of disgust, which is known to weaken with exposure (i.e., Rozin, 2008). If administered after the task, any ratings might have been affected by habituation from experiencing them in the scanner. Thus, we utilized an independent group of participants to establish the vignette characteristics.

We predicted the following basic differences between these groups of vignettes: (1) the moral disgust vignettes should be more disgusting and reflect greater purity violations than the moral anger vignettes, but the moral anger vignettes should reflect greater justice violations and be at least equivalent or more in terms of their ability to induce anger; (2) that the matched disgust vignettes should not be judged to reflect moral violations and that they should principally induce disgust, but that they should do so to a lesser extent than the high disgust vignettes; and (3) that the neutral and scrambled vignettes should not arouse much emotional feeling.

### Materials and methods

#### Participants

Twenty undergraduate participants (M age = 22.2, *SD* = 2.8), nine male, participated for course credit or a small cash payment. This research was approved by the Macquarie University Human Research Ethics Committee. Specifically, all participants gave their written informed consent and all procedures of the study (including the written informed consent, and the autonomy of each participant to stop at any point during the study) were kept in line with the MQ HREC regulations.

#### Stimuli

The vignettes are presented in Table 1.

#### Procedure

Participants were presented with each of the vignettes in Table 1 in a different random order. For each vignette, participants were asked to rate how angry, happy, fearful, sad, and disgusted it made them feel, in each case using seven-point category scales (1 [Not at all] to 7 [Very]). In addition, for all vignettes except the neutral and scrambled ones, participants also rated: (1) How immoral is this scenario? (1 [Not at all immoral] to 7 [Very immoral]); (2) How likely is it that a person is directly responsible for the events in this scenario? (1[Not at all likely] to 7 [Very likely]); (3) How wrong is the behavior in this scenario? (1[Not at all wrong] to 7 [Very wrong]); (4) How well does this behavior fit the following definition “Purity—Behaviors in this category are felt to be unclean, unnatural, and contaminated or polluted” (1 [Not at all] to 7 [Very well]); (5) How well does this behavior fit the following definition “Harm—Behaviors in this category are felt to cause suffering or pain to others” (1 [Not at all] to 7 [Very well]); and (6) How well does this behavior fit the following definition “Justice—Behaviors in this category are felt to create inequality, be unfair, or otherwise restrict others' rights” (1 [Not at all] to 7 [Very well]). These ratings were always presented in the same order. Once all of the vignettes had been rated (participants were allowed to stop and rest as needed) the task was complete.

#### Analysis

Participants' scores for each vignette were averaged and the unit of analysis was the vignette. Only the moral disgust, moral anger, matched disgust, and high disgust vignette groups were compared, as the control scores for neutral and scrambled vignettes had almost no variance with means (excepting the happiness rating) all around the lowest point on the rating scale. Each rating type was analyzed using a univariate ANOVA, with vignette group treated as a between participant factor. *Post-hoc* testing was conducted with Ryan-Einot-Gabriel-Welsch (REGW) Range tests. This test is recommended to describe homogenous subsets of means (Howell, 1997).

### Results

All of the ratings, by vignette group, are presented in Table 2, alongside the *post-hoc* contrasts conducted for each of the univariate ANOVAs described below.

*Disgust*. Univariate ANOVA revealed a significant effect of Vignette group, *F*_(3, 76)_ = 19.40, MSE = 0.48, *p* < 0.001, partial-eta squared = 0.43. A *post-hoc* REGW Range test revealed three homogenous subsets, the matched disgust and moral anger vignettes as one, the high disgust as the next, and the moral disgust vignette group as the highest scorer on this variable.
*Anger*. Univariate ANOVA revealed a significant effect of Vignette group, *F*_(3, 76)_ = 33.90, MSE = 0.98, *p* < 0.001, partial-eta squared = 0.57. A *post-hoc* REGW Range test revealed two homogenous subsets, the matched disgust and high disgust vignettes as one, and the moral disgust and moral anger vignette groups as the other and highest scoring subset on this variable.
*Fear*. Univariate ANOVA revealed a significant effect of Vignette group, *F*_(3, 76)_ = 10.85, MSE = 0.43, *p* < 0.001, partial-eta squared = 0.30. A *post-hoc* REGW Range test revealed two homogenous subsets, the matched disgust vignettes as one, and the remaining three vignette groups as the other and highest scorer on this variable.
*Sad*. Univariate ANOVA revealed a significant effect of Vignette group, *F*_(3, 76)_ = 22.66, MSE = 0.41, *p* < 0.001, partial-eta squared = 0.47. A *post-hoc* REGW Range test revealed three homogenous subsets, the matched disgust group of vignettes as one, the high disgust vignette group as the next, and the moral anger and moral disgust vignettes as the third and highest scoring subset on this variable.
*Happy*. Univariate ANOVA revealed a significant effect of Vignette group, *F*_(3, 76)_ = 5.14, MSE = 0.14, *p* < 0.01, partial-eta squared = 0.17. There were no homogenous subsets.
*Immoral*. Univariate ANOVA revealed a significant effect of Vignette group, *F*_(3, 76)_ = 39.39, MSE = 0.89, *p* < 0.001, partial-eta squared = 0.61. A *post-hoc* REGW Range test revealed two homogenous subsets, the matched disgust and high disgust vignettes as one, and the moral disgust and moral anger vignette groups as the other and highest scorer on this variable.
*Responsibility*. Univariate ANOVA revealed a significant effect of Vignette group, *F*_(3, 76)_ = 20.96, MSE = 0.85, *p* < 0.001, partial-eta squared = 0.45. A *post-hoc* REGW Range test revealed two homogenous subsets, the matched disgust and high disgust vignettes as one, and the moral disgust and moral anger vignette groups as the other and highest scoring subset on this variable.
*Wrong*. Univariate ANOVA revealed a significant effect of Vignette group, *F*_(3, 76)_ = 30.86, MSE = 1.03, *p* < 0.001, partial-eta squared = 0.55. A *post-hoc* REGW Range test revealed two homogenous subsets, the matched disgust and high disgust vignettes as one, and the moral disgust and moral anger vignette groups as the other and highest scorer on this variable.
*Purity*. Univariate ANOVA revealed a significant effect of Vignette group, *F*_(3, 76)_ = 23.16, MSE = 0.72, *p* < 0.001, partial-eta squared = 0.48. A *post-hoc* REGW Range test revealed two homogenous subsets, the moral disgust vignette group as the first and highest scoring subset on this variable, with the remaining three vignette groups as the other subset.
*Harm*. Univariate ANOVA revealed a significant effect of Vignette group, *F*_(3, 76)_ = 26.27, MSE = 0.91, *p* < 0.001, partial-eta squared = 0.51. A *post-hoc* REGW Range test revealed two homogenous subsets, the matched disgust and high disgust vignettes as one, and the moral disgust and anger vignette groups as the other and highest scorer on this variable.
*Justice*. Univariate ANOVA revealed a significant effect of Vignette group, *F*_(3, 76)_ = 45.44, MSE = 0.74, *p* < 0.001, partial-eta squared = 0.64. A *post-hoc* REGW Range test revealed three homogenous subsets, the moral anger vignette group as the first and highest scoring subset of this variable, the moral disgust vignette group as the second, and the matched and high disgust vignette groups as the third.

**Table 2:** Characteristics (Mean and SD) of the vignette groups (Moral, Matched and High Disgust, Moral Anger, Neutral and Scrambled, respectively) evaluated in the pilot study and used in the main study.

**Measure**	**Vignette grouping**					
	**Moral disgust**	**Matched disgust**	**High disgust**	**Moral anger**	**Neutral**	**Scrambled**
Disgust	5.6 (0.8)_c_	4.1 (0.7)_a_	4.9 (0.7)_b_	4.3 (0.5)_a_	1.0 (0.0)	1.2 (0.1)
Anger	5.0 (1.1)_b_	2.8 (0.8)_a_	3.1 (1.3)_a_	5.3 (0.7)_b_	1.0 (0.0)	1.4 (0.1)
Fear	3.4 (0.8)_b_	2.3 (0.5)_a_	3.0 (0.6)_b_	3.0 (0.6)_b_	1.1 (0.1)	1.2 (0.1)
Sad	3.6 (0.6)_c_	2.3 (0.5)_a_	3.0 (0.7)_b_	3.9 (0.7)_c_	1.1 (0.0)	1.2 (0.0)
Happy	1.9 (0.5)_a_	1.7 (0.4)_a_	1.5 (0.1)_a_	1.9 (0.3)_a_	3.4 (0.4)	2.6 (0.2)
Immoral	5.1 (1.1)_b_	2.8 (0.6)_a_	3.0 (1.2)_a_	5.3 (0.8)_b_		
Responsible	6.0 (0.6)_b_	4.4 (1.2)_a_	4.2 (1.1)_a_	5.9 (0.5)_b_		
Wrong	5.4 (1.1)_b_	3.2 (0.8)_a_	3.4 (1.3)_a_	5.5 (0.9)_b_		
Purity	5.6 (0.8)_a_	4.0 (0.9)_b_	4.3 (1.1)_b_	3.5 (0.5)_b_		
Harm	5.0 (1.2)_a_	3.1 (0.8)_b_	3.3 (1.0)_b_	5.2 (0.7)_a_		
Justice	4.0 (1.0)_b_	2.5 (0.6)_c_	2.5 (0.9)_c_	5.2 (0.9)_a_		

*For each measure, variables with different letters significantly differed from each other (Neutral and Scrambled were not compared)*.

^*^Each measure was compared across vignette groupings.

### Discussion

The results from the pilot study confirmed that the vignettes were able to induce the requisite emotions and reactions in participants that we intended to investigate in the main study. The moral disgust vignettes were more disgusting and reflected greater purity violations than the moral anger vignettes, which in turn scored higher on justice violations but equivalent in anger to the moral disgust vignettes. The matched disgust vignettes were significantly less disgusting than the high disgust vignettes, and both these sets of vignettes were judged to be less immoral and wrong compared to the moral anger and disgust vignettes. Perhaps most importantly, for our purposes, the matched disgust vignettes, which contained *identical* core elicitors to the moral disgust vignettes, were judged to be less disgusting, suggesting that participants regarded the addition of a degree of intentionality (i.e., responsibility, on which the moral disgust vignettes scored higher than the matched disgust vignettes) as making these scenarios more disgusting, more anger provoking, and as involving greater purity and harm violations.

That each of the vignette types generated a range of emotions is not itself a major issue. The dominant emotion in each case was consistent with our categorization of the vignettes. Indeed, most publications on this topic find that other emotions are activated beyond the target specific ones (e.g., Gutierrez and Giner-Sorolla, 2007; Russell and Giner-Sorolla, 2011; Case et al., 2012; Olatunji et al., 2016). Thus on the basis of self-report, participants felt that an immoral action involving a core disgust elicitor was more disgusting than the core elicitor without an immoral action. This suggests that, at least for self-report accounts, the presence of the moral violation generates *additional* disgust—in line with the term “moral disgust.” This finding is consistent with the view that our “moral center” is linked to the area of our brain in which physical revulsion—core disgust—is situated (Moll et al., 2005; Schaich Borg et al., 2008; Schnall et al., 2008). This idea was examined more directly in the main study where fMRI was used to investigate differences between moral and core disgust, rather than relying on self-report accounts.

## Main study

We used functional magnetic resonance imaging (fMRI) to measure the blood-oxygen level-dependent (BOLD) response in participants reading the various vignettes tested in the pilot. The purposes of the main study were: (1) to determine if the brain areas activated by the matched disgust vignettes were largely the same or different from those produced by the moral disgust vignettes, as both used the same set of core disgust elicitors, but the latter involved moral violations and the former did not; (2) to see if brain activation induced by moral disgust vignettes more closely resembles that of the matched or the high disgust vignettes, on the basis that self-report disgust scores were closer in the pilot study for moral and high disgust, than for moral and matched disgust; (3) to test if activation differences between the high and moral disgust conditions were similar to activation differences between the matched and high disgust condition. In both cases, the first mentioned set of vignettes groupings (i.e., high vs. moral and matched vs. high) were less disgusting than the second set, and we can use the brain activation to assess whether common neural networks are implicated; and (4) to examine if activation induced by the moral disgust vignettes more closely resembled that of the anger vignettes than that of the matched and high disgust vignettes, on the basis that self-report anger scores were closer in the pilot study for moral disgust and moral anger.

### Materials and methods

#### Participants

Twenty-two healthy undergraduate participants (M age = 26.3, *SD* = 5.6), 10 male, successfully completed the study (two were lost due to movement artifacts). They were awarded course credit or received a small cash payment. All participants provided written informed consent and were recruited on the basis of the following (self-reported) inclusion criteria: (1) healthy; (2) neurologically normal (validated by neuroradiological review); (3) non-healthcare employed; (4) between the ages of 18 and 30 years; and (5) are able to undergo fMRI scanning (i.e., no metal implants, pacemaker, claustrophobia, etc). No pilot participants were involved in the main study. All procedures of the study (including the written informed consent, and the autonomy of each participant to stop at any point during the study) were in line with the MQ HREC regulations and the study protocol was approved by this committee.

#### Stimuli

Participants were presented with the vignettes (validated in the pilot work and developed uniquely for this study) on a monitor viewed via a mirror mounted on the headcoil to read while being scanned. Participants were instructed to indicate (button-press) whenever a scrambled vignette appeared. The task was simply to ensure that participants were attending to the statements. The vignettes are presented in Table 1.

#### fMRI procedure

We used an event-related design with six runs. Each run involved presenting 55 vignettes. Of these 55, there were 10 vignettes drawn from each of the five conditions (moral disgust, matched disgust, high disgust, moral anger, neutral). A further five scrambled vignettes were randomly interpolated amongst these trials. Vignettes could only be selected once on each run from the moral disgust, matched disgust, high disgust, and moral anger conditions (i.e., random sampling without replacement), while random sampling with replacement was used for the neutral and scrambled vignettes. Each vignette was presented for a total of 6 s, with no inter-stimulus interval. Participants were asked to press a response button whenever a scrambled vignette appeared. The presentation of stimuli was controlled using Presentation software (Neurobehavioral Systems, Albany, CA, USA; http://www.neurobs.com/). The vignettes were presented using a 15-inch Macintosh Power Book and projected onto a screen positioned ~1.2 m behind the participant's head. The participants viewed the screen via a mirror mounted ~15 cm above the eyes.

The MRI data were acquired using a Siemens Verio 3T scanner using a 32-channel head coil (Erlangen, Germany) at Macquarie Medical Imaging, Macquarie University Hospital, Sydney, Australia. Gradient echo T2^*^-weighted echo planar imaging (EPI) was implemented for functional imaging. One scan volume was obtained every 4 s (TR; hence stimulus presentation was jittered with respect to the scanner) and consisted of 49 slices (TE = 40 ms, FOV = 240 x 240 mm, in-plane resolution 2.0 x 2.0 mm, slice thickness 2.4 mm, interslice gap 0.5 mm, flip angle = 90°). The first four volumes in each run were automatically discarded. Each run comprised 84 recorded volumes.

For each participant a high-resolution structural image was acquired (3D-MPRAGE sequence, voxel size 0.94 isotropic, FOV: 240 x 240 mm, 176 slices, TR = 2110 ms, TE = 3.54 ms, flip angle = 9°).

#### fMRI analysis

The data were analyzed with SPM8 (Wellcome Department of Cognitive Neurology, London, U.K., https://www.fil.ion.ucl.ac.uk/spm/) using standard pre-processing steps. First, all obtained volumes per participant were realigned and resliced to correct for small head-movements and slice timing correction was performed. The mean of the functional volumes was co-registered, segmented and normalized to an MNI (Montreal Neurological Institute) template within SPM. The obtained transformation parameters were then applied to the co-registered functional volumes, which were re-sampled to a 2 x 2 x 2 mm voxel size. The spatially transformed functional data were spatially smoothed using a 6 mm FWHM isotropic Gaussian kernel. A general linear model (GLM) was fitted to the data with five regressors. Each condition was modeled with a boxcar function and convolved with SPM's canonical hemodynamic response function. Furthermore, to remove low-frequency drifts, the default temporal high-pass data filter cut-off (128 s) was employed.

A general linear model (GLM) was estimated for each participant with one regressor for each of the six conditions: five conditions of interest (moral disgust, matched disgust, high disgust, moral anger, neutral) and the scrambled vignettes. The events were modeled using an event-related function lasting from the onset of each event to the onset of the following event. After fitting the GLMs, the scrambled condition was subtracted from each of the five conditions of interest (i.e., condition minus scrambled) and the resulting beta estimates were then entered into the second-level analysis.

We conducted whole brain analyses using a repeated measures design and performed with a threshold of *p* < 0.001 and a cluster threshold of 10 contiguous voxels (Leiberman and Cunningham, 2009), which effectively counteracts the increased Type I error rate from multiple comparisons by only taking as significant clusters of 10 or more voxels that have activation above the threshold. Anatomical labeling was done using WFU_Pickatlas on any clusters that reached significance.

### Results

#### Disgust vs. neutral control vignettes

Table 3 presents the clusters and regional maxima for the three contrasts examining high, matched, and moral disgust conditions against the neutral control vignettes (each of these first having the activation in the scrambled condition subtracted; i.e., [High Disgust-Scrambled]>[Neutral-Scrambled]; [Matched Disgust-Scrambled]>[Neutral-Scrambled]; [Moral Disgust-Scrambled]>[Neutral-Scrambled]). All three contrasts revealed activity in inferior, middle and superior frontal gyri, around similar coordinates. Similarly, activation occurred in the right mid-temporal and left fusiform gyri, in all three contrasts. Fewer activations were observed for the moral and matched disgust contrasts in the occipital gyrus and for high disgust contrast in the superior and middle temporal gyri. Unique regional activations were largely absent on the left side.

**Table 3:** Exploratory analyses of disgust and control related contrasts.

	**High disgust > Neutral regional maxima (MNI)**						**Matched disgust > Neutral regional maxima (MNI)**						**Moral disgust > Neutral regional maxima (MNI)**					
***Region***	***x***	***y***	***z***	***T***	***Ke***	***Side***	***x***	***y***	***z***	***t***	***Ke***	***Side***	***x***	***y***	***z***	***t***	***Ke***	***Side***
**Fusiform Gyrus**	**−38**	**−44**	**−18**	**7.61**	**1876**	**L**	**−38**	**−40**	**−18**	**7.37**	**594**	**L**	**−38**	**−40**	**−18**	**6.89**	**539**	**L**
	**−38**	**−16**	**−30**	**3.96**	**14**	**L**	**−40**	**−16**	**−32**	**4.18**	**15**	**L**	**−40**	**−14**	**−32**	**3.96**	**13**	**L**
*Lingual Gyrus*	*12*	*−68*	*−8*	*6.37*	*1132*	*R*							*−22*	*−76*	*−8*	*4.03*	*48*	*L*
													*8*	*−80*	*−8*	*4.01*	*73*	*L*
Supramarginal Gyrus	−52	−56	26	6.16	737	L												
**Medial Frontal Gyrus**	**−12**	**46**	**30**	**5.59**	**537**	**L**	**−4**	**52**	**−18**	**5.3**	**148**	**L**	**−2**	**50**	**−18**	**4.96**	**215**	**L**
**Inferior Frontal Gyrus**	**−36**	**34**	**−16**	**5.49**	**242**	**L**	**−50**	**16**	**16**	**6.1**	**1906**	**L**						
							**42**	**30**	**−8**	**4.62**	**626**	**R**	**44**	**30**	**−6**	**4.69**	**154**	**R**
*Middle Frontal Gyrus*	*−40*	*2*	*58*	*3.47*	*13*	*L*	*−32*	*4*	*44*	*5.17*	*441*	*L*						
**Superior Frontal Gyrus**	**−6**	**14**	**68**	**3.35**	**11**	**L**	**−10**	**50**	**30**	**4.62**	**653**	**L**	**−8**	**50**	**30**	**6.36**	**1076**	**L**
							**12**	**24**	**58**	**3.74**	**13**	**R**						
*Uncus*	*−32*	*−2*	*−36*	*5.46*	*47*	*L*							*26*	*0*	*−32*	*3.72*	*10*	*R*
*Parahippocampa Gyrus*	*−20*	*−4*	*−16*	*4.7*	*172*	*L*	*−18*	*−6*	*−18*	*4.06*	*16*	*L*						
	*24*	*−2*	*−18*	*4.35*	*41*	*R*												
*Lentiform Nucleus*	*−26*	*−8*	*0*	*4.58*	*22*	*L*							*−18*	*6*	*4*	*3.9*	*56*	*L*
	*28*	*−6*	*2*	*3.89*	*11*	*R*							*18*	*4*	*0*	*3.83*	*10*	*R*
	*−20*	*4*	*4*	*3.84*	*12*	*L*												
*Uvula*	*22*	*−72*	*−34*	*4.36*	*109*	*R*	*22*	*−72*	*−34*	*4.03*	*64*	*R*						
**Sub–Gyral**	**−24**	**−80**	**−2**	**4.34**	**80**	**L**	**−32**	**10**	**28**	**3.89**	**25**	**L**	**−32**	**0**	**44**	**3.76**	**19**	**L**
	**−34**	**30**	**12**	**7.16**	**876**	**L**												
	**−32**	**2**	**42**	**3.97**	**36**	**L**												
	**18**	**−18**	**46**	**4.23**	**15**	**R**												
**Middle Temporal Gyrus**	**38**	**−6**	**−36**	**4.14**	**11**	**R**	**54**	**6**	**−16**	**3.62**	**11**	**R**	**56**	**4**	**−28**	**3.7**	**29**	**R**
							**−50**	**−18**	**−8**	**5.59**	**2434**	**L**	**42**	**12**	**−36**	**3.69**	**16**	**R**
							**−48**	**0**	**−24**	**3.69**	**13**	**L**						
*Superior Temporal Gyrus*							*52*	*−40*	*4*	*3.84*	*48*	*R*	*−56*	*−28*	*−2*	*6.69*	*4794*	*L*
													*54*	*8*	*−16*	*3.97*	*13*	*R*
Extra–Nuclear	−28	8	−4	3.99	18	L												
Middle Occipital Gyrus	−40	−84	0	3.9	18	L												
	38	−88	−2	3.59	16	R												
Superior Occipital Gyrus	−34	−88	22	3.62	24	L												
*Cuneus*	*14*	*−80*	*32*	*3.85*	*41*	*R*							*16*	*−78*	*2*	*3.69*	*15*	*R*
Postcentral Gyrus	62	−12	20	3.75	18	R												
*Precuneus*							*−2*	*−54*	*34*	*3.66*	*44*	*L*	*−46*	*−2*	*48*	*3.76*	*32*	*L*
Pyramis													0	−52	32	5.23	311	R
Precentral Gyrus													22	−74	−36	4.83	135	R

*Bold type indicates that the same brain area is active across all three disgust conditions of the experiment, the italic type indicates that the same brain area is active in two disgust conditions of the experiment. Plain type means the brain area activation is unique to that disgust condition*.

In the right hemisphere, there was both less activity than on the left and more limited overlap amongst the three contrasts. Activation in the frontal inferior gyri was observed for the matched and moral disgust vignettes, lentiform nucleus activation for the high and moral disgust vignettes. There was more unique regional activity on the right side, but this was not clearly confined to one particular type of vignette. In sum, the three disgust-related vignettes, moral, matched, and high, produced similar and overlapping patterns of activation, characterized by left frontal regions and the fusiform gyrus.

#### Comparing the disgust-related vignettes

**Figure 1:**
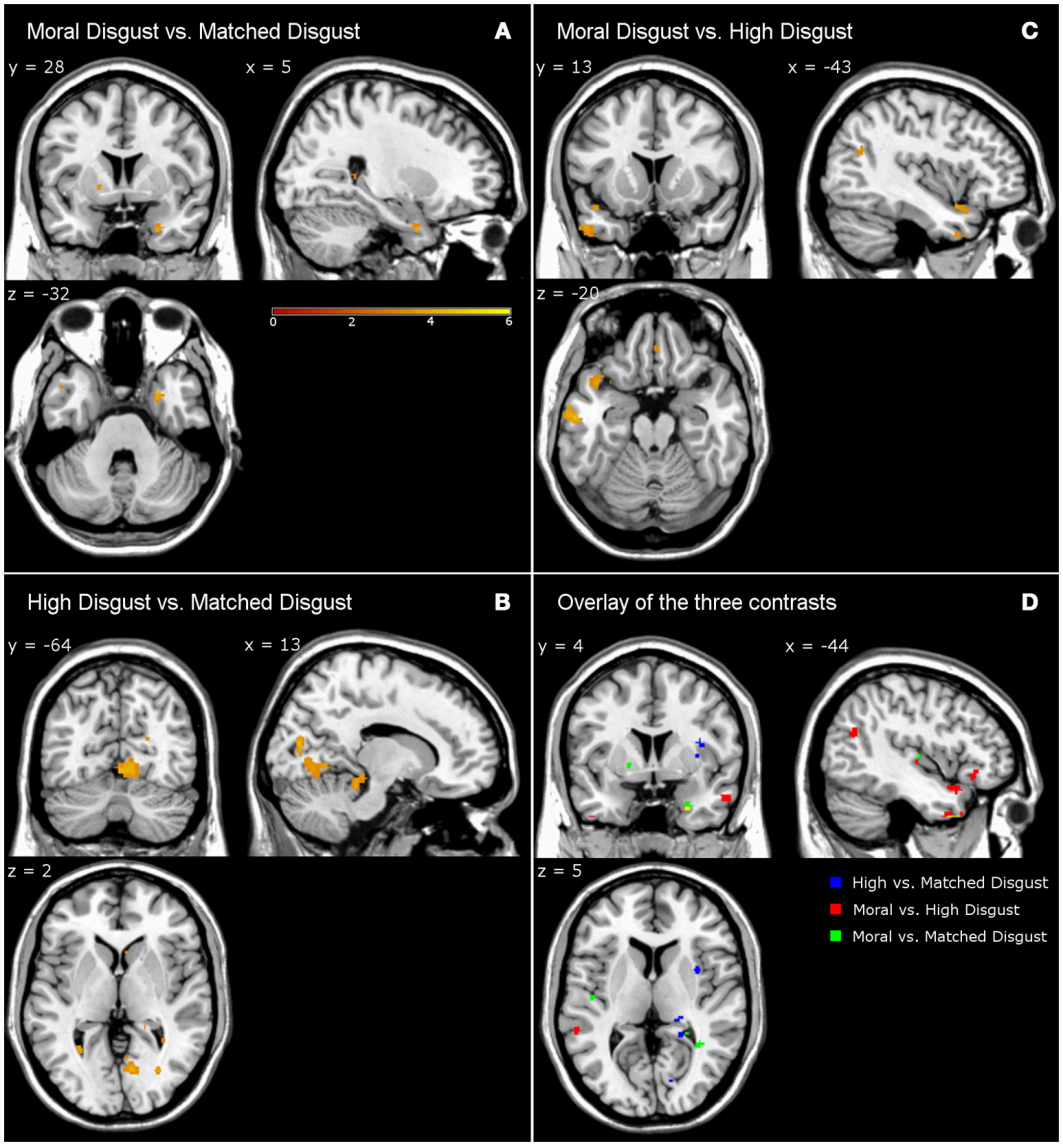
Contrasts from fMRI data. Moral disgust vs. matched disgust contrast **(A)**, high disgust vs. matched disgust contrast **(B)**, moral disgust vs. high disgust contrast **(C)**, and overlay of the three disgust contrasts **(D)**. Different to Legend color (i.e., yellow) in lower right panel shows the overlap of the three disgust contrasts. Legend shows T-value of the contrasts (e.g., higher T-value represents stronger activations).

Table 4 presents the activations following high-matched, moral-high, and moral-matched disgust contrasts. Overall, there were far fewer similarities between vignette types than observed in the disgust-neutral contrasts presented in Table 3. Figure 1 illustrates that for the moral-matched disgust contrast there were few activations, with these mainly on the right, and restricted to the uncus, culmen, and one sub-gyral location. On the left, the only activation was for the middle temporal gyrus, similar to an activation observed for moral anger (see **Table 6**). Thus, after controlling for matched disgust, the moral component alone was associated with little unique neural activity.

**Table 4:** Exploratory analyses of disgust-related contrasts.

	**High disgust > Matched disgust regional maxima (MNI)**						**Moral disgust > High disgust regional maxima (MNI)**						**Moral disgust > Matched disgust regional maxima (MNI)**					
***Region***	***x***	***y***	***z***	***T***	***Ke***	***Side***	***x***	***y***	***z***	***t***	***Ke***	***Side***	***x***	***y***	***z***	***t***	***Ke***	***Side***
Inferior Frontal Gyrus							−46	28	−12	3.6	13	L						
							48	28	−8	3.59	15	R						
Inferior Temporal Gyrus							−58	−12	−20	4.63	132	L						
*Middle Temporal Gyrus*							*−46*	*6*	*−40*	*4.37*	*78*	*L*	*−46*	*8*	*−40*	*3.67*	*14*	*L*
							*−52*	*−40*	*2*	*3.87*	*51*	*L*						
							*−44*	*−64*	*24*	*3.85*	*29*	*L*						
							*58*	*4*	*−26*	*3.71*	*19*	*R*						
Superior Temporal Gyrus							48	18	−30	3.66	20	R						
							−42	14	−20	3.79	41	L						
Middle Occipital Gyrus	30	−72	2	4.11	10	R												
Precuneus							2	−52	36	4.26	120	R						
Cuneus	10	−80	14	4.79	261	R												
Rectal Gyrus							4	38	−22	4.02	23	R						
Uncus													26	6	−32	4.45	16	R
*Sub–Gyral*	*−34*	*30*	*12*	*4.94*	*40*	*L*							*36*	*−50*	*6*	*3.98*	*11*	*R*
	*−30*	*−54*	*2*	*3.86*	*17*	*L*												
	*20*	*−70*	*20*	*4.06*	*26*	*R*												
	*18*	*−36*	*0*	*3.95*	*26*	*R*												
	*34*	*−46*	*0*	*3.9*	*10*	*R*												
Parahippocampa Gyrus													34	−8	−24	3.86	16	R
*Culmen*	*14*	*−36*	*−16*	*4.02*	*42*	*R*							*14*	*−36*	*−16*	*3.65*	*16*	*R*
Culmen of Vermis	4	−64	−6	4.84	353	R												
Insula	38	6	12	4.08	40	R												

*Bold type indicates that the same brain area is active across all three disgust conditions of the experiment, the italic type indicates that the same brain area is active in two disgust conditions of the experiment. Plain type means the brain area activation is unique to that disgust condition*.

Participants in the pilot study judged both the moral and high disgust vignettes as more disgusting than the matched disgust vignettes. In the neural data as depicted in Figure 1, subtracting matched from high disgust ([High-Scrambled]>[Matched-Scrambled]) revealed significant clusters in several regions including the middle occipital gyrus, cuneus, culmen, and insula, as well as sub-gyrally. Apart from the culmen, these regions were not active in the analogous contrast for moral disgust ([Moral-Scrambled]>[Matched-Scrambled])—see Table 4. Thus, although both vignette types were perceived as more disgusting than the matched vignettes by participants in the pilot, the pattern of brain activation notably differed, including significant insula activation in the high-matched contrast. This suggests a different neural basis for disgust reported for core elicitors relative to disgust reported for moral disgust elicitors.

We also contrasted the moral and high disgust vignettes directly [Moral-Scrambled]>[High-Scrambled]). Figure 1 shows significant clusters in the left inferior frontal gyrus, inferior temporal gyrus, and middle and superior temporal gyri. Interestingly, anger-related activations (see **Table 6**) were predominantly characterized by extensive left-middle temporal activations. Recall that the pilot data indicated that the moral disgust vignettes were more disgusting than the high disgust vignettes, and that the high disgust vignettes were more disgusting than the matched disgust vignettes—both to the same extent (see Table 2). So while these two conditions differed in disgust to the same extent in the pilot, Figure 1 shows there was no overlap in neural activations when they are contrasted in the fMRI data. In other words, the brain areas associated with differences in one were not the same as those associated with the other. This suggests again that comparable differences in reported disgust between comparisons involving core elicitors (i.e., high disgust > matched disgust) are supported by different neural structures to those involving moral disgust (i.e., moral disgust > high disgust).

Note that all the brain images follow the neurological convention (i.e., the left side of the image corresponds to the patient's left side of the brain and vice versa).

#### Anger and disgust

**Figure 2:**
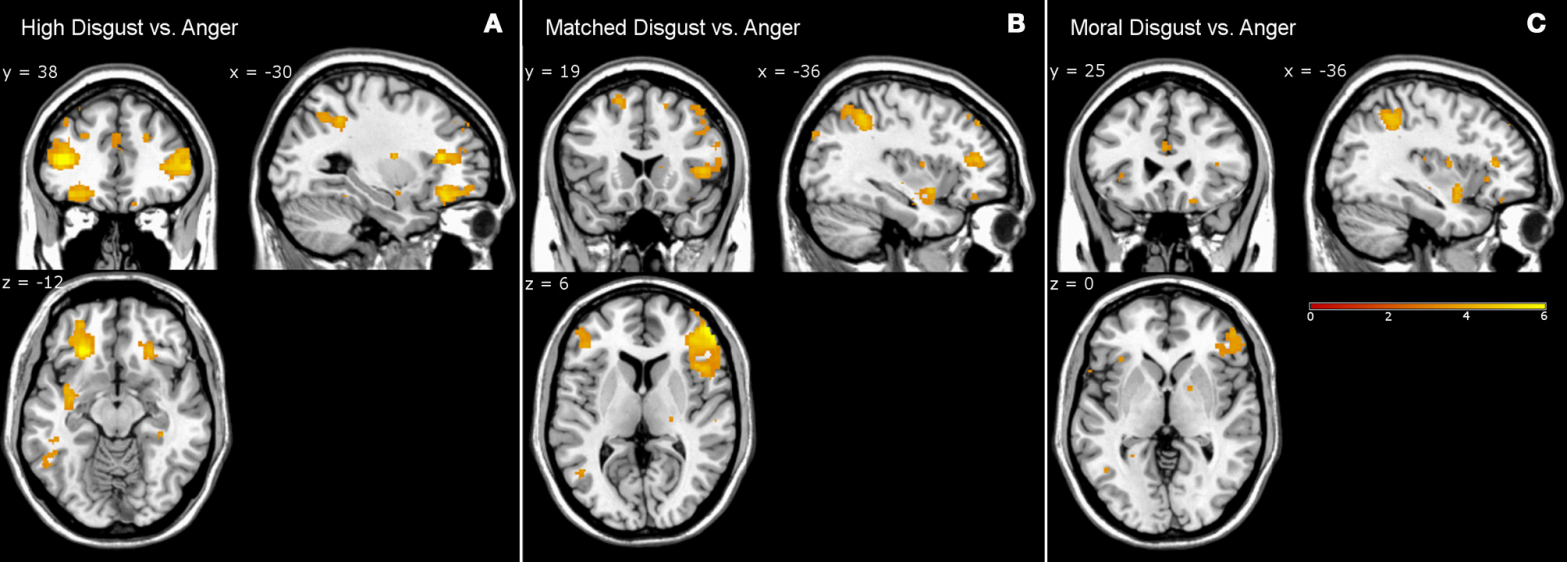
Contrasts from fMRI data. High disgust vs. anger contrast **(A)**, matched disgust vs. anger contrast **(B)**, and moral disgust vs. anger contrast **(C)**.

Table 5 presents the high disgust, matched disgust and moral disgust contrasts minus anger. Two things are apparent when viewing Table 5. First, there are numerous activations for all three contrasts, indicating fairly major patterns of difference in the brain activations associated with disgust and those associated with anger. Second, right-sided differences are far more apparent here than in the various disgust-related contrasts examined so far. Predominantly, activity in two regions disambiguated moral anger and disgust-related responses. First, for both the left and right sides, there was extensive activation of inferior, and middle frontal gyri. Second, on both sides, frontal activations covered considerably larger regions for both the high disgust minus anger contrast (see Figure 2, left panel) and the matched disgust minus anger contrast (see Figure 2, middle panel), than for moral disgust anger contrast (see Figure 2, right panel). In sum, relative to moral anger, the matched, high and moral disgust vignettes produced overlapping activations (i.e., broad qualitative similarity), but the regional size of these activated regions differed between moral disgust and the two other disgust-related conditions (i.e., a broad quantitative difference).

**Table 5:** Exploratory analyses of disgust-related contrasts.

	**High disgust**> **Anger regional maxima(MNI)**						**Matched disgust**>**Anger regional maxima(MNI)**						**Moral disgust**>**Anger regional maxima(MNI)**					
***Region***	***x***	***y***	***z***	***T***	***Ke***	***Side***	***x***	***y***	***z***	***t***	***Ke***	***Side***	***x***	***y***	***z***	***t***	***Ke***	***Side***
**Inferior Frontal Gyrus**	**−38**	**32**	**12**	**8.75**	**1143**	**L**	**−42**	**34**	**12**	**5.53**	**669**	**L**	**−38**	**32**	**10**	**4.52**	**103**	**L**
	**52**	**42**	**6**	**6.59**	**871**	**R**	**−32**	**14**	**−20**	**4**	**20**	**L**	**−32**	**14**	**−20**	**3.89**	**24**	**L**
							**52**	**46**	**4**	**7.32**	**2102**	**R**	**54**	**42**	**6**	**4.73**	**333**	**R**
							**36**	**16**	**−16**	**4.14**	**37**	**R**						
**Middle Frontal Gyrus**	**−22**	**30**	**−18**	**8.64**	**556**	**L**	**−22**	**28**	**−18**	**6.67**	**374**	**L**	**−22**	**28**	**−20**	**4.35**	**97**	**L**
	**−38**	**40**	**40**	**4.3**	**39**	**L**	**−18**	**16**	**60**	**4.46**	**85**	**L**	**20**	**26**	**−18**	**4.16**	**66**	**R**
	**22**	**28**	**−18**	**6.04**	**215**	**R**	**−24**	**54**	**14**	**3.85**	**36**	**L**						
							**22**	**26**	**−18**	**5.5**	**213**	**R**						
**Inferior Parietal Lobule**	**−56**	**−36**	**40**	**6.11**	**1293**	**L**	**40**	**6**	**38**	**3.96**	**257**	**R**	**−66**	**−32**	**28**	**3.84**	**26**	**L**
													**36**	**−50**	**44**	**4.19**	**93**	**R**
**Middle Temporal Gyrus**	**−48**	**−54**	**−4**	**5.63**	**459**	**L**	**44**	**−50**	**50**	**5.45**	**1043**	**R**	**−48**	**−54**	**−2**	**3.53**	**14**	**L**
	**54**	**−52**	**−4**	**4.25**	**85**	**R**	**−50**	**−54**	**−2**	**4.7**	**323**	**L**						
							**54**	**−52**	**−4**	**4.7**	**260**	**R**						
Superior Occipital Gyrus							−36	−86	32	4.19	20	L						
							−42	−84	24	3.58	10	L						
*Fusiform Gyrus*	*50*	*−48*	*−20*	*4.03*	*20*	*R*	*−48*	*−38*	*−16*	*3.85*	*13*	*L*						
*Parahippocampa Gyrus*	*−24*	*−2*	*−20*	*4*	*35*	*L*							*−22*	*−4*	*−20*	*4.07*	*22*	*L*
	*−34*	*−26*	*−20*	*3.71*	*14*	*L*												
Supramarginal Gyrus	58	−42	38	4.44	237	R												
*Cingulate Gyrus*	*−8*	*−30*	*38*	*3.93*	*53*	*L*	*6*	*−32*	*28*	*3.96*	*12*	*R*						
*Anterior Cingulate*	*−6*	*26*	*−4*	*3.86*	*41*	*L*							*0*	*24*	*24*	*3.94*	*24*	*R*
Posterior Cingulate	8	−32	26	5.26	85	R												
*Uncus*	*28*	*6*	*−26*	*4.04*	*18*	*R*							*26*	*6*	*−32*	*5.17*	*35*	*R*
Culmen	2	−62	−8	3.73	18	R												
**Extra–Nuclear**	**−32**	**−6**	**14**	**4.91**	**39**	**L**	**−38**	**6**	**−10**	**4.88**	**114**	**L**	**−38**	**4**	**−8**	**4.97**	**104**	**L**
	**8**	**24**	**14**	**4.49**	**370**	**R**	**−30**	**−6**	**14**	**3.93**	**20**	**L**	**−32**	**−4**	**12**	**4.48**	**34**	**L**
	**38**	**−26**	**0**	**3.71**	**10**	**R**	**36**	**−4**	**−8**	**3.68**	**19**	**R**						
	**30**	**−40**	**8**	**3.68**	**13**	**R**												
	**38**	**10**	**−8**	**3.62**	**11**	**R**												
*LateralVentricle*	*2*	*2*	*6*	*3.55*	*15*	*R*							*28*	*−42*	*6*	*4.07*	*12*	*R*
													*10*	*−12*	*20*	*3.87*	*19*	*R*
**Sub–Gyral**	**−36**	**−8**	**−12**	**4.6**	**253**	**L**	**−32**	**−48**	**40**	**5.24**	**903**	**L**	**−32**	**−46**	**42**	**4.97**	**546**	**L**
	**−26**	**−50**	**0**	**4.22**	**22**	**L**	**−26**	**−48**	**0**	**4.43**	**16**	**L**	**34**	**−10**	**−12**	**3.59**	**17**	**R**
	**−46**	**−36**	**−14**	**3.97**	**16**	**L**	**−42**	**8**	**20**	**3.92**	**15**	**L**						
	**−42**	**6**	**20**	**3.8**	**33**	**L**	**28**	**−56**	**26**	**3.89**	**10**	**R**						
	**24**	**38**	**28**	**4.03**	**72**	**R**												
	**34**	**−34**	**−12**	**3.97**	**12**	**R**												

*Bold type indicates that the same brain area is active across all three disgust conditions of the experiment, the italic type indicates that the same brain area is active in two disgust conditions of the experiment. Plain type means the brain area activation is unique to that disgust condition*.

Finally, we also conducted a contrast of anger vs. the neutral vignettes, which is presented in Table 6. Here, the most predominant activation by region size was in the left middle temporal gyrus, followed by left sided activations in the inferior, medial frontal gyri. Several other activations were present, including lingual and fusiform gyri, cuneus and parahippocampal gyri.

**Table 6:** Exploratory analysis of the anger-neutral contrast.

	**Anger > Neutral regional maxima (MNI)**					
***Region***	***x***	***y***	***z***	***t***	***Ke***	***Side***
Medial Frontal Gyrus	−8	48	30	6.34	973	L
	−6	48	−16	5.82	379	L
Inferior Frontal Gyrus	−48	16	16	4.92	215	L
Middle Temporal Gyrus	−60	−4	−16	8.38	4657	L
Superior Temporal Gyrus	54	8	−16	5.23	525	R
	42	−50	20	3.79	27	R
Precuneus	−4	−52	34	6.94	667	L
Lingual Gyrus	8	−80	−8	6.44	2497	R
Fusiform Gyrus	−38	−42	−20	6.12	413	L
	−40	−14	−32	4.68	41	L
Precentral Gyrus	−50	−2	50	4.9	193	L
Uvula	20	−74	−34	4.53	186	R
Lentiform Nucleus	−20	4	4	4.1	29	L
Cuneus	16	−82	28	3.97	33	R
Parahippocampa Gyrus	−24	−6	−30	3.83	16	L

### Discussion

The basic finding from the main study is that brain activations generated by moral disgust inducing vignettes closely resemble activations generated by disgust inducing vignettes. Although at first glance this might seem to confirm the existence of a “moral” disgust, our carefully matched disgust condition, which used the same set of core elicitors but without any moral violations, shows this is not the case. Contrasts between moral, matched and high disgust vignettes did not suggest that moral disgust vignettes generate “additional disgust” as reported by participants in the pilot study (i.e., moral > high > matched disgust—see Table 2). First, where the magnitude of disgust is manipulated by choosing more potent core elicitors (high disgust > matched), the resulting pattern of activation is different to that generated by the moral and matched disgust contrast (moral > matched), which also differed in magnitude of reported disgust but used the same core disgust elicitors. Thus, whatever drives higher disgust ratings for high disgust > matched is unlikely to be the same factor that drives higher disgust ratings for moral vs. matched disgust. Second, for the high disgust > matched disgust and for the moral > high disgust contrasts there were no common activations—and again where differences in the magnitude of reported disgust were evidenced in the pilot—there was no overlap in activations, suggesting once more that whatever neural structure/s drive higher disgust ratings for moral disgust elicitors these do not overlap with those driving higher disgust ratings for core disgust elicitors. Third, the comparison of the moral and high disgust conditions and the moral and matched disgust conditions both revealed activation differences that seemed to reflect a moral-anger related component to moral disgust, with common activation in the middle temporal lobe. Fourth, that after controlling for matched disgust, moral disgust is associated

with little unique neural activation. Fifth, comparisons of the disgust-related conditions with moral anger suggest that while there are qualitative similarities in activation patterns shared by all of the disgust conditions, there are quantitative differences, with the relationship to moral anger being different for moral disgust relative to matched and high disgust.

## General discussion

This study is the first investigation of whether core and moral disgusts entrain common neural systems using appropriate controls for immoral events with references to core disgust. The present results suggest that: (i) activation of overlapping brain regions between core and moral is the result of content overlap in the vignettes—*core disgust elicitors*—across conditions, and not from moral violations *per se*, and (ii) moral “residue”—the effect remaining once core disgust is taken into account—produces activation that is more consistent with moral anger than one of “additional disgust.”

These findings call attention to a number of issues. First, there is a dissociation between the self-report data and the fMRI data. On the basis of the self-report data alone, we would be led to believe that an immoral action involving a core disgust elicitor was more disgusting than a core elicitor without an immoral action. The implication of this is that the presence of the moral violation ramped up experienced disgust—establishing *moral* disgust. However, the objective fMRI data suggests otherwise: there is no evidence that moral disgust evokes greater activation in “disgust areas” activated by core elicitors. Consequently, we believe this delivers compelling evidence against the idea that moral violations can themselves be disgusting in the same way that core elicitors can be. Indeed, dissecting the nature of this presumed relationship is of increasing interest (Curtis, 2011). For example, one important question is whether disgust started by expanding from its origins in distaste, to serving as a pathogen avoidance mechanism, before finally entering into the social sphere (Tybur et al., 2009). A further question is whether the disgust-morality relationship is purely metaphorical (Nabi, 2002)? Or, is disgust evoked solely because moral violations include core disgust stimuli such as, for example, sexual crimes and bloody murders (Royzman and Sabini, 2001; Oaten et al., 2009). The evidence from the current study favors the latter account. To our knowledge, this is the only study to control for the influence of core disgust referents contained in moral disgust vignettes, and our findings indicated that the moral disgust vignettes did not produce a disgust-like pattern of activation *after* the influence of core disgust referents were accounted for (i.e., moral disgust—matched disgust). Moreover, the residual activation from the moral disgust vignettes was comparable to moral anger, rather than disgust.

This finding is especially interesting. A number of researchers have argued that verbal reports of disgust in response to moral transgressions are problematic due to the usage of the term “*disgusting*” in colloquial language to describe events that are angering or irritating (Chapman et al., 2009), which is why we did not ask participants to make disgust ratings in our study. Indeed, Nabi (2002) reported that the lay understanding of the word “*disgust*” corresponds more closely to the theoretical meaning of anger, than to that of disgust. Specifically, events recalled in response to the words “*disgust*” and “*disgusted*” tended to reflect primarily anger-related themes (e.g., demeaning offenses) rather than classic disgust-related themes (e.g., noxious elements). This claim is also supported by past studies that report the words “*disgust*” and “*disgusted*” evoke feelings associated with anger-related concepts (e.g., Roseman et al., 1994; Rozin et al., 1999). Therefore, it might be that the dissociation between the self-reported and fMRI data in the present study is an artifact of the data collection method. That is, participants may be relying on lay meanings of disgust—e.g., reflecting greater anger than repulsion—when appraising moral violations in the vignettes and are consequently misattributing the label of disgust to their experienced affect (Royzman et al., 2014), a suggestion that is consistent with our fMRI data.

Second, the data reported here challenge the view that the insula serves as the seat of disgust processing. A large area of the left frontal regions and the fusiform gyrus was identified in the analysis, but it did not extend into the insular cortex as in previous studies of disgust (Phillips et al., 1997, 1998, 2004; Calder, 2003; Krolak-Salmon et al., 2003; Wright et al., 2004)—although importantly, insula activation was present when high disgust was contrasted with matched disgust (see Table 5), suggesting that this structure may be one of those involved (along with the others identified in the contrast in Table 4) in mediating increases in emotional intensity for disgust. Nonetheless, the results from the disgust-neutral contrasts, which reveal activations unique to this emotional state (rather than say the difference from another state such as anger), are consistent with a growing number of studies showing that the insula is often, but not always involved in disgust processing (Phillips et al., 1998; Stark et al., 2003, 2005). Moreover, our findings are also consistent with a meta-analysis of imaging studies that found the anterior insula to be no more active during disgust than other emotions (Wager et al., 2007). We note in passing that the method of inducing disgust could be one variable that influences the activation profile, including the presence of insula activity, and could explain some of the variability between studies. This work involves the first fMRI investigation of whether core and moral disgusts entrain common neural systems, using immoral vignettes with appropriate controls for referents to core disgust, and our findings suggest that separate neural systems mediate these reactions.

## Limitations

It is important to acknowledge that the processing of disgusting stimuli is likely influenced by individual differences (i.e., disgust sensitivity; Haidt et al., 1994), and the fact that these data were not collected is a limitation of the current study. Consequently, it is not possible to exclude potential participant differences in trait disgust across the two studies (i.e., pilot study vs. main study).

## Conclusion

Our results support two clear inferences. First, that presenting moral violations that include core disgust elicitors will activate very similar brain regions to those activated by presenting the same core elicitors alone without moral violations. Thus in the absence of a matched disgust condition as used here, one could falsely conclude that a unique moral disgust existed, when in fact most of this moral disgust actually results from the presence of core disgust elicitors referred to in the vignettes, not from the moral violations *per se*. Second, examination of the moral residue (i.e., contrast of moral disgust with matched disgust or high disgust) provides no clear evidence that it induces a disgust-like pattern of activation in the brain. Rather, this residue seems somewhat more akin to the pattern produced by moral anger. Although the participants in the pilot study rated the moral disgust vignettes as more disgusting than both the matched and high disgust vignettes, this additional disgust does not appear to rely on the same neural correlates that generate stimulus-driven differences in disgust (e.g., seeing dog feces vs. stepping in it barefoot).

## Author contributions

MO, RS, and TC conceived of the study. MO, RS, MW, and AR designed the study. MO and RS coordinated the study. MO, RS, MW, and AR administered study. MW, MB, and RS participated in data and statistical analysis, all authors helped draft the manuscript, and gave final approval for publication.

### Conflict of interest statement

The authors declare that the research was conducted in the absence of any commercial or financial relationships that could be construed as a potential conflict of interest.
